# Nanofiller Reinforced Biodegradable PLA/PHA Composites: Current Status and Future Trends

**DOI:** 10.3390/polym10050505

**Published:** 2018-05-07

**Authors:** Jingyao Sun, Jingjing Shen, Shoukai Chen, Merideth A. Cooper, Hongbo Fu, Daming Wu, Zhaogang Yang

**Affiliations:** 1 College of Mechanical and Electrical Engineering, Beijing University of Chemical Technology, Beijing 100029, China; sunjingyao5566@sina.com (J.S.); yutoujuzi@gmail.com (S.C.); andrewlinxy@gmail.com (H.F.); 2 School of Civil Engineering & Architecture, Taizhou University, Taizhou 318000, Zhejiang, China; syusejing@gmail.com; 3 Department of Chemical and Biomolecular Engineering, The Ohio State University, Columbus, OH 43210, USA; cooper.1774@buckeyemail.osu.edu; 4 State Key Laboratory of Organic-Inorganic Composites, Beijing 100029, China

**Keywords:** biodegradable, nanocellulose, carbon nanotubes, nanoclay, polymer composites

## Abstract

The increasing demand for environmental protection has led to the rapid development of greener and biodegradable polymers, whose creation provided new challenges and opportunities for the advancement of nanomaterial science. Biodegradable polymer materials and even nanofillers (e.g., natural fibers) are important because of their application in greener industries. Polymers that can be degraded naturally play an important role in solving public hazards of polymer materials and maintaining ecological balance. The inherent shortcomings of some biodegradable polymers such as weak mechanical properties, narrow processing windows, and low electrical and thermal properties can be overcome by composites reinforced with various nanofillers. These biodegradable polymer composites have wide-ranging applications in different areas based on their large surface area and greater aspect ratio. Moreover, the polymer composites that exploit the synergistic effect between the nanofiller and the biodegradable polymer matrix can lead to enhanced properties while still meeting the environmental requirement. In this paper, a broad review on recent advances in the research and development of nanofiller reinforced biodegradable polymer composites that are used in various applications, including electronics, packing materials, and biomedical uses, is presented. We further present information about different kinds of nanofillers, biodegradable polymer matrixes, and their composites with specific concern to our daily applications.

## 1. Introduction

With the depletion of energy and the worsening environmental problems associated with plastic scrap disposal from petroleum production, there has been a growing interest in biodegradable and renewable resources. With the increasing demand for biodegradable materials, the advancements in the field of degradable composites are getting more and more attention [1,2,3,4,5,6]. However, most commercial composites are carbon fiber or fiberglass-reinforced epoxy composites that are the subject of fossil-fuel-based composites and are not in line with sustainable green development because of the difficulties in recovering and disposing of these materials at their life-cycle end [7,8,9,10,11]. At the same time, traditional biodegradable polymer materials exhibit poor mechanical properties, a narrow processing window, low electrical and thermal properties and cannot meet the actual needs [12,13,14]. However, using biodegradable polymer materials as substrates, biodegradable polymer composites that can both degrade and meet practical requirements can be developed by adding appropriate reinforcing fillers using advanced technologies and methods. Biodegradable composite materials are used in many fields such as artificial joints, wound treatment, delivery of corresponding drugs and body orthopedic devices, and are widely used in food packaging and agricultural films [15,16,17]. To expand the range of applications of biodegradable polymers in various fields, the performance of biodegradable polymers needs to be enhanced. An example of this is the use of bio-based hybrid nanocomposites that would enhance the synergy of natural fibers in bio-based polymers while improving performance and maintaining environmental attractiveness [1,7,18]. With the changing environment, people are becoming increasingly integral to waste treatment. With the biodegradability of bio-based polymers, sustainability is also enhanced so that they can be applied in practical engineering. The rapid development of bio-based polymers is due to the significant progress in the production of bio-based components from biomass feedstocks, enabling bio-based polymers with more functional and chemical structures to achieve target performance and functionality. However, the mechanical properties and barrier properties of biopolymers limit their popularization and application [19]. There are three aspects related to the promotion of biopolymers: performance, processing and cost. All biodegradable polymers have “performance and processing” issues. Nano-enhanced biodegradable biomaterials can be used to improve the performance of biomaterials. Bio-nanocomposites are comprised of biopolymer matrices and nanoparticles (sizes less than several hundred nanometers) which are used for the reinforcement or functionalization [20,21,22]. With the high depth to width ratio and high superficial area of nanoparticles, bio-nanocomposites are a new type of material that has significantly improved performance compared to the basic bio-polymers [23,24,25]. In the packaging industry, the continuous impact of plastomer packaging castoff on the environment has also attracted worldwide attention due to limited processing methods [26,27,28,29].

Thus, the biodegradable plastic packaging receives more attentions attribute to its sustainable consumption of natural resources while the environmental burden is becoming more and more serious [30,31]. At the same time, the increase of food security led to an increased need for biodegradable wrappers made from renewable resources (biomacromolecules), as a substitute for synthetic plastic wrappers, particularly for short period wrapping and single-use (i.e., single-use tableware, single-use dishes, glass and tableware, garbage bags, drink vessels, farm mulching films, fast food boxes, medical apparatus and instruments, etc.) [25]. From the current situations, the application of nanotechnology in packaging is much faster than the food itself, because people are suspicious of the safety of nanoparticles that are directly added to the food. For food packaging materials, it is believed that nanoparticles will not affect the ingredients of the food itself. It will only migrate from packaging to the food under high temperature level and longer heating time. Therefore, the biodegradable nanocomposites used as packaging materials have minimum effect on the food security [32,33,34]. Prior to mercantile use of bio-based wrappers, some elementary problems must be solved including the rate of degradation, changes in mechanical behavior during conservation, possibility of microbial proliferation, and contamination of packaged foods by harmful compounds. In fact, these biopolymer wrappers have a higher hydrophilicity and are weaker during processing, resulting in industrial restrictions [35]. Although researchers have done much research on improving the packaging performance of biopolymer membranes, the issues of physics, thermology, and mechanical behavior still cannot meet the requirements in industrial activities [31,36]. Consequently, we have been working on developing biocomposites with improved mechanical behavior, separation properties, rheology and hot properties of food packaging pellicles. There are many bio-based polymer fillers available. The nature of the nanofiller can be organic or inorganic. For example, inorganic fillers include silicon dioxide (SiO_2_), titanium dioxide (TiO_2_), calciumcarbonate (CaCO_3_), polyhedral oligomeric silsesquioxane (POSS) and other particles. Coconut shell nanofillers, cellulose nanofillers, and other organic and natural derivatives belong to the class of organic fillers [37,38,39,40,41]. Different nanofillers can improve the mechanical properties, heat resistance, barrier properties and can promote the development of biodegradable materials [6,42,43,44].

This article summarizes the latest research progress of biologic macromolecule materials, nanofillers and bio-nanocomposites in hardgoods, packing, electron and biomedical applications. Bio-based composites can overcome the inherent disadvantages of some bio-based materials such as narrow processing windows, poor barrier properties, poor biocompatibility and conductivity. This article also looks forward to the future processing technology, product development and application of nanofillers to enhance biocomposite materials [45].

## 2. Biodegradable Polymers

Biobased polymers [46,47,48,49] can be categorized into: (1) upgrades of biodegradable polylactic acid (PLA), polyhydroxyalkanoates (PHA), and the like; (2) polymerization similar to fossil oil derived materials, for instance bio aggregation (Bio-PET); and (3) new biologically based polymers, for instance 2,5- furan two formate (PEF) [50]. Polymer materials from natural crops, including corn-based isosorbide polycarbonate, can also be regarded as biologically based polymers. This article generally describes two biopolymers of polylactic acid (PLA) and polyhydroxyalkanoates (PHA). PLA, PHA and butanedioic acid polymers are the general biologically based polymers, which are often used in biodegradable plastic operations for the addressing of environmental problems. With the changing social demand for biodegradable polymers, the performance needs to be improved to make it more applicable [51,52]. Recently, examples of the development and application of PLA, PHA and succinate polymers have been described. The basic characteristics and chemical properties of the nanocomposites made from PLA, PHA, and some other polymers are also introduced in the following section.

### 2.1. Biodegradable Polylactic Acid (PLA)

PLA is made from the ring opening polymerization (ROP) of the ring two polymer lactide from lactic acid [53]. The nexus among the nature of PLA and the content of the L unit was studied. Polylactic acid is receiving increasing attention due to its biodegradability and potential role for replacing traditional polymers. PLA belongs to a highly crystalline polymerization because it is stereotactic. These stereoisomers can be controlled by using diverse activators. As the first large-scale produced bio-based plastic, PLA has good mechanical properties, mainly by hydrolytic degradation [54,55,56].

**Figure 1:**
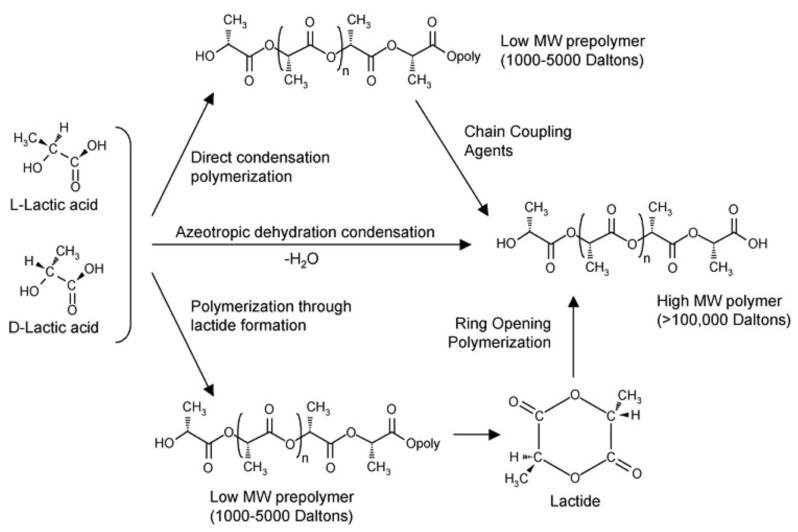
Synthesis of PLA from l- and d-lactic acids. Reproduced with permission from [57].

Since PLA’s raw materials are based on agricultural raw materials, the continuous supply of PLA resins is of great significance to the development of the global agricultural economy. The increase in the high molecular weight of polylactic acid is the driving force for the extended application of PLA. Various techniques can be used to prepare these polymerizate, mainly through the formation of azeotropic dewatering polycondensation, direct polycondensation, or condensation of the lactide (as shown in Figure 1) [57]. Although the production technology of PLA has been greatly improved, there are still many areas for improvement in PLA applications. PLA has mainly been used to replace thermoplastics. For example, for important foods that require high separate safeguard, polyethylene terephthalate (PET) packaging may not be replaced by PLA, because the barrier properties of PLA are different from those of PET. The brittleness of PLA will also be a limiting condition for its tenacity and shock resistance. Finally, when PLA is exposed to atrocious weather conditions, it may have unpredictable characteristics. Compared to conventional thermoplastic polymers, polylactic acid has poor heat endurance and shock resistance. As a result, there is a gap between PLA and conventional polymers. However, the use of polylactic acid could be extensive if its performance was improved. In recent years, researchers have used a variety of nanofillers to improve the performance of PLA, mainly phyllosilicates, carbon nanotubes, hydroxyapatite, layered titanates, etc. [58]. The good compatibility, material characteristics and low cost of PLA will make it widely used in medicine. Furthermore, the shear-thinning characteristics of PLA can be used in conventional polymer processing technology processes, and some new technologies (such as the use of supercritical foams and electrospinning to produce nanofibers) which has positive implications for spreading the application of such polymers [59].

### 2.2. Polyhydroxyalkanoate (PHA)

Polyhydroxy chain alkanoate (PHA) is a kind of biological polyester, made naturally by various microorganisms. Research work is being done to create PHA in transgenic plants [60]. These polymeric compounds have different structures and corresponding properties. More than 150 different genres of PHA are homopolymers, which are created by diverse kinds of bacteria and growing conditions. Polyhydroxybutyrate (PHB) and poly (hydroxybutyrate Hydroxyvalerate) (PHBV) are the most famous polymerizates in polyhydroxyalkanes systems [54]. PHA is a member of the polyester family composed of hydroxyalkanate monomers. In nature, they exist in the form of homopolymers. PHA is present in pure particulate polymer form and is used as an energy storage medium in bacteria (similar to animal fats and starch-like starch plants). PHA is commercially produced using energy-rich feedstocks that are converted to fatty acid feedstocks. During PHA’s industrial production process, the cells are separated and dissolved after several production cycles. The extraction of the purified cell residue from a polymer is processed into particles or powders [61].

**Figure 2:**
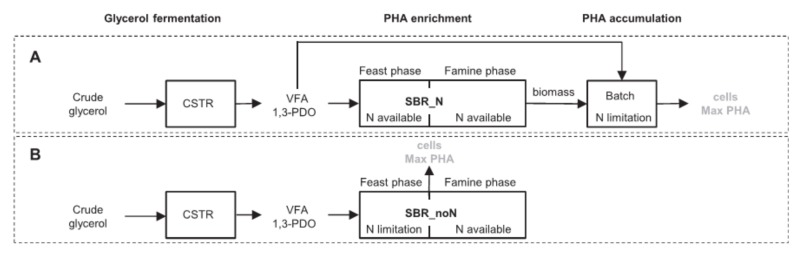
Summary of strategies for production of PHA from fermented crude glycerol Reproduced with permission from [68].

In recent years, researchers found that a large number of bacteria can produce different polyhydroxyalkanoate bio-polyesters. In general, it does not seem too easy to manipulate PHA structure and the proportion of monomers in the copolymer. However, the weakening of the beta oxidation cycle by pseudomonad putida and pseudomonad thermophila makes various PHA structures controllable. It has become a reality to use functionalized PHA by introducing functional groups, which contains fatty acids, into polyhydroxy polymer chains in a predetermined ratio [62]. However, commercialization of PHA is costly, has molecular weight (MW) and structural instability, and, thus, the thermomechanical properties are not stable [36,63,64]. Furthermore, high costs are associated with complex biological processes such as bactericidal effects, low conversion of carbon substrates to PHA products, slow growth of microorganisms, and downstream separation [65]. Therefore, researchers are not aware of the limitations of PHA production. A growing body of research has been conducted on methods to enhance production, with the objective being sustainably producing large-scale microbial polyhydroxyalkanoates (PHAs), promoting the commercialization of PHAs and expanding their range of applications [66,67]. For example, Burniol-Figols summarized the method for the production of PHA by fermenting crude glycerol (as shown in Figure 2) and studied the effects of nitrogen under different conditions [68]. Koller focuses on the investigation of the thermal and rheological properties of PHA polymers accumulated by *Synechocystis salina*. The determined thermal and rheological properties show that PHA polymers accumulated by *S. salina* on digestate supernatant or mineral medium are comparable with the commercial available poly(3-hydroxybutyrate). However, the results demonstrated that PHA polymers generally need to be modified before the melting process to increase their stability in the molten state [69].

PHA is not only a kind of environmentally friendly biopolymer, but also has many adjustable material properties. With the further reduction in cost and the development of high value-added applications, it will become a kind of multi-application field material which can be accepted by the market. Because it is a family with a wide range of components, its performance from hard to high flexibility enables it to be applied to different applications. The structural diversity of PHA and the variability of its properties make it an important member of biomaterials. Compared with PLA, the developing history of PHA is short, but the development potential and range of applications are bigger.

## 3. Nanofillers for Biodegradable Nanocomposites

Nanofillers can improve or adjust the properties of the materials into which they are incorporated, such as flame retardant properties, optical or electrical properties, mechanical properties and thermal properties [57]. Nanofillers need to be incorporated into the polymer matrix in a certain proportion. There are many nanofillers used in nanocomposites, which mainly include nanoclays, carbon nanotubes and some organic nanofillers [54]. This paper mainly introduces nanofiller reinforced biodegradable polymer composites, in which the matrix need to come from renewable resources.

### 3.1. Nanocellulose

**Figure 3:**
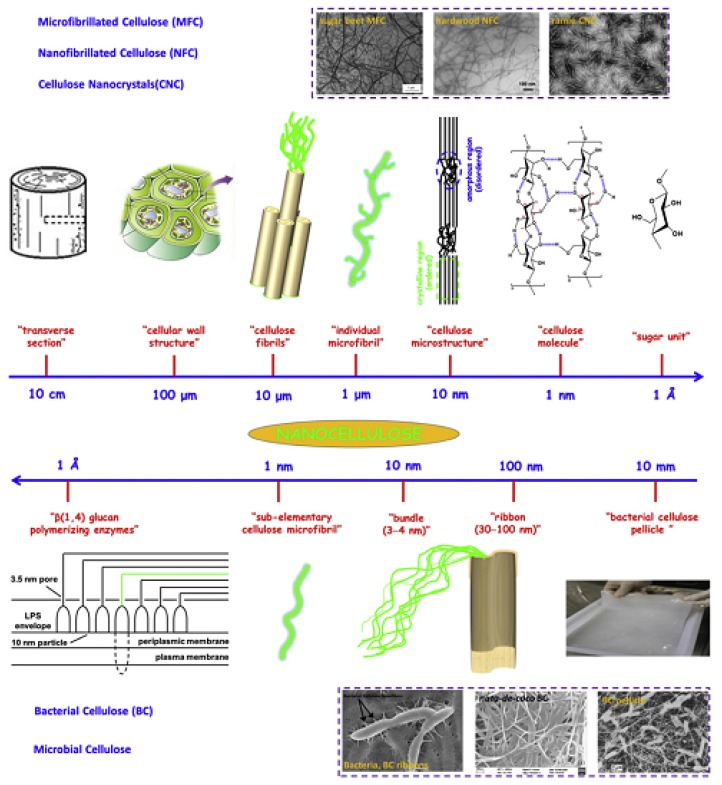
Hierarchical structure of cellulose; top image (from large unit to small unit): cellulose nanocrystals (CNC), micro/nanofibrillated cellulose (MFC and NFC); bottom image (from tiny unit to small unit): bacterial cellulose (BC). Reproduced with permission from [72].

With the advancement of technology, the natural polymer cellulose attracts more people’s attention worldwide. A new kind of “cellulose” has been used as an advanced material [70]. Cellulose is considered a product or extract of natural cellulose consisting of nanoscale structural materials. In general, the cellulose family can be divided into three kinds: (1) cellulose nanocrystals which has other names such as nanocrystalline cellulose; (2) cellulose nanofibers which is also known as nanofibrillated cellulose (NFC); and (3) bacterial cellulose (BC), also called microbial cellulose. It can be obtained from wood, flour, beets, potato tubers, ramie, algae and other plants. The BC can be reproduced quickly by converting large unit (cm) into small units (nm) and let them grow back into large units under adapt circumstance. Bacterial nanocellulose is a nanocellulose that is secreted by microorganisms and has been demonstrated to be useful for artificial blood vessels in tissue engineering. Bacterial nanocellulose as nanofiller has good mechanical properties and biocompatibility, ultrafine fiber network and high porosity [71]. Chemically induced deconstruction strategies, such as acid hydrolysis, are commonly applied to pick up CNC from natural cellulose while retaining highly crystalline structures. The process of BC construction is shown in Figure 3, which is typically synthesized by bacteria in a pure form [72]. Different types of cellulose exhibit different properties, which determine their applicability and functionality, that is, some types of cellulose are better suited for specific applications. A high Young’s modulus/high tensile strength are the typical properties of cellulose that are important. Some aspect ratios that can be manipulated depend on particle type, and potential compatibility with other materials. In addition, the choice of chemistry and material affinity give rise to very versatile cleavage [73]. There are many studies on nanofibers. Jafari et al. [74] conducted a study of nanofiber coatings and found that the low DE of polymer–polymer complexes decreases the water adsorption and the solubility of maltodextrin. Second, the crystalline nanocellulose fibers increase the path of curvature and curvature in the material and reduce the water possibility of molecular penetration. Kuo et al. developed an enhanced bio-nanocomposite fiber/resin interface with a blend of toughened epoxy resins to improve resin penetration and fiber distribution [75]. Nanocellulose can control the rheology stability of dispersion and give the composite stronger mechanical properties. Synthetic modification of fibrous cellulose is a way to get chemical compatibility of the systems. However, this also limits the environmental benefits of using cellulose components. Therefore, an attractive step forward in compatibility and further expansion of nanocellulose applications is through the use of surfactants [76]. The tensile strengths of some nanocomposites are linearly correlated with the strengths of cellulose nanofibers. Better dispersion of individual cellulose nanofibers can improve the performance of the composites [77].

Linear biopolymer cellulose is naturally found in all plants. In addition to be the major natural polymer on Earth, it has a variety of functions, including excellent biocompatibility, lower density, greater strength, and has the most favorable mechanical properties at a fraction of the cost. With the recyclability, anisotropic shape, good mechanical properties, fine biocompatibility, and adjustable surface chemistry of nanocellulose, a growing number of applications of nanocellulose materials in science and biomedical engineering related fields has attracted peoples’ interest. Although the topics of nanocellulose has been extensively studied over the past years, there is clear room for growth, especially regarding new developments in coatings and medical devices [73,78].

### 3.2. Nanoclays

Layered silicate, also known as clay, is the most commonly used nanolayered silicate nanocomposite in polymer composites, which have a widespread application in the preparation of composites based on clay. The changes in size of the silicate layer depends on the source of the clay, the silicate particles and the production technology. The structure in the silicate layer will be changed, and the substitute induces a negative charge in the slime sheet that is spontaneously offset by the cations in the interlayer spacing. All electricity variation relies on layered silicate [79]. Biodegradable plastic clay nanocomposites have attracted wide attention because of their ameliorated mechanical and obstruction properties and their lower burnable points on each native polymer. Majeed researched the natural fiber filled hybrid composite in the field of food applications and obtained a wrapper with enhanced barrier and obstruction properties [80]. Malwela studied the enzymatic degradation behavior of nanometer clay reinforced degradation material. The effect of nanoclay on the degradation rate of blends at various temperatures is diverse from that of PLA/PBSA mixture composites, which provide a reference for subsequent studies. Of course, nanoclays are also used for several new and improved biodegradable polymer materials, with a growing range of applications, not only because of its strong practicality and low price point, but also because it has machinability and thermosetting properties [81,82,83,84,85].

### 3.3. Carbon Nanotubes and Graphene

#### 3.3.1. Carbon Nanotubes

Nanotubes have been synthesized by a number of methods including arc discharge, laser ablation, chemical vapor deposition, etc. [86]. Carbon nanotubes exhibit excellent mechanical, electrical and magnetic properties which make them an ideal material for high-strength polymer composites. However, because of van der Waals interactions, carbon nanotubes usually form stable bundles that are extremely hard to disperse and align in the polymer. Due to this, the biggest issue with the manufacturing of carbon nanotube-reinforced composites is the ability to disperse and assess the dispersibility as well as arranging and controlling the carbon nanotubes in the matrix. There are some methods for dispersing nanotubes in a polymer matrix, such as solution mixing and melt mixing. The chemical vapor deposition process has progressed the construction of carbon nanotubes, which has promoted the widespread industrial application of this process.

Carbon nanotubes can be used in many situations, including polymer composites, electrochemical energy storage/conversion, hydrogen storage and others. Polymer composites are used as functional fillers that not only increase the thermal/mechanical properties, but also provide additional functions such as increase in flame resistance, and barrier properties. Its polymer composites have been extensively studied in many fields. Carbon nanotubes have been effectively exploited by researchers to develop techniques based on renewable resources, polymer materials, etc. Ma et al. [87] proposed the design of a bio-based conducting and rapidly-crystallizing nanocomposite with controlled distribution of multi-walled carbon nanotubes through an interface stereocomplex. Thin films and (semi) conductive materials show significant potential.

Hapuarachchi and co-worker [88] found that the multi-walled carbon nanotubes can be applied as flame retardants for PLA and its natural fiber reinforced composites. They found that the heat release rate (HRR) was reduced by 58% compared to native PLA because of the addition of multi-walled carbon nanotubes. The advancement of carbon nanotubes with better conductivity has created new applications for polymer composites in electromagnetic interference shielding fields.

#### 3.3.2. Graphene

Graphene is a single layer of hybridized carbon atoms arranged in a two-dimensional lattice which can be manufactured by the peeling off of graphite nanosheets. The theoretical specific surface area of graphene sheets is 2630–2956 square meters and the aspect ratio is more than 2000. With its special structure, graphene has outstanding thermal properties and mechanical properties. One of the most advanced applications of graphene is a filler for nanocomposite polymers. However, it is hindered by poor solubility in most cases. Moreover, the large surface area of graphene leads to significant aggregation in the polymer because of the van der Waals. Thus, graphene has significant optical activity that can be observed on some substrates by simple optical methods. Actually, different numbers of atomic layers of graphene can be distinguished relatively easily using transmission optical microscopy [89].

Due to the excellent electrical, mechanical, optical and transport properties of graphene, it has been used in many different types of applications. Graphene reinforced nanocomposites have a high level of hardness and strength. These nanocomposites should have excellent mechanical properties and graphene is expected to be a reinforcing material for high performance nanocomposites. However, there is a problem in obtaining good dispersion, and there are challenges in getting graphene to completely peel into a single layer or a few layers of material having a reasonable lateral size or generating graphene oxide without significantly damages [90]. Researchers have done a lot of work on graphene reinforced nanocomposites. Graphene-based adsorbents have attracted widespread interest as effective adsorbents for the removal of heavy metals from the environment [91]. Sima Kashi et al. conducted a study on the dielectric and EMI shielding performance of graphene-based biodegradable nanocomposites. The study found that the addition of graphene nanosheets significantly enhanced the dielectric constant of both polymers [92]. Purnima Baruah et al. studied bio-based, tough, hyperbranched epoxy/graphene (HBE) nanocomposites with enhanced biodegradability. Performance studies showed that the addition of graphene oxide (GO) to HBE increased the bond strength by 189%, the toughness by 263%, the tensile strength by 161%, and the elongation at break by 159% [93]. After extensive research, it has also been necessary to guarantee that a strong interface exists between the reinforcing material and the polymer matrix to give the best properties of the graphene reinforced nano-polymer. It should be noted that apart from providing good prospects for mechanical enhancement, it can also be used to control functional properties such as conductivity, swelling, gas barrier properties and stability.

### 3.4. Other Functional Nanofillers

Bio-nanoparticles and a variety of functional fillers are attracting a great deal of attention because of their diverse biomedical and biotechnological applications. Nano-scale fillers play a significant role in the manufacture of biological composites because they bring a variety of desirable functions to the composite. There are a variety of functional nanofillers such as silica nanoparticles, hydroxyapatite, layered double hydroxide (LDH), polyhedral oligomeric silsesquioxanes (POSS), cellulose nanofibers, etc. [94,95]. Recently, some nanofillers have drawn more attention due to their versatility in the manufacture of biomedical applications. Hydroxyapatite is famous for its bioactive and biocompatible ceramic which is found in bones and teeth. Bio-based polymers of LDH find wide applications in tissue engineering, drug delivery and gene therapy because of their compatibility and their non-cytotoxic and nonirritating biological systems [96]. The Hap/GO nanocomposites prepared by M. Ramadas et al. provide excellent biocompatibility for use in orthopedic, drug delivery and dental applications [97].

## 4. Processing Methods and Applications

### 4.1. Processing Methods

Bio-based materials have disadvantages such as poor hydrophilicity, poor electrical conductivity, and poor mechanical properties during processing [21]. However, nanofillers can overcome the above defects and achieve the purpose of enhancing the properties of composite materials. Therefore, nanofillers are used to enhance biodegradable materials. There are many ways to prepare nanofiller biodegradable composite materials. Different nanofillers have different treatment methods. This article describes the processing of nanocellulose based biocomposites, the preparation of nanoclay based composites, the processing of polymer–carbon nanotube based biocomposites, and the processing of functional nanocomposites.

#### 4.1.1. Processing of Nanocellulose Based Bio-Nanocomposites

At present, the main treatment methods for using nanocellulose fillers are solvent casting and melt processing [98]. These are mainly used to solve the problem that the nanocellulose cannot be evenly dispersed in a non-polar medium. Due to the polarity of nanocellulose whiskers, it is difficult to disperse homogeneously in non-polar media and therefore needs to be uniformly dispersed in polar media or in aqueous media [99]. Solvent casting and melt processing methods can help nanocellulose evenly disperse in the polymer. For the above two methods, the polymer used is different. For solvent casting, mainly three types of polymers are used: (1) water-soluble polymer; (2) polymer emulsion; and (3) water-insoluble polymer. There are two effective ways to achieve solvent casting. For polymer emulsions and water-insoluble polymers, it is possible to utilize polar head and long hydrophobic tail surfactants of polymer emulsions or water-insoluble polymers, Surfactants are coated on the surface of nanocellulose crystals. Another method is to graft hydrophobic chains onto the surface of nanocellulose crystals. Both methods allow the nanocellulose filler to be uniformly dispersed in the polymer.

Melt extrusion is the most commonly used in industry [100]. Melt extrusion refers to the process of adding the plasticized material to the extruder for forming. However, this method has several tough problems. The biggest problem currently is the use of dry nanocellulose. During the extrusion process, the nanocellulose particles easily form hydrogen bonds in the amorphous state. These hydrogen bonds have a strong adsorption force, which makes the material prone to aggregate when it is dry, so it is difficult to evenly disperse the polymer. Currently, researchers are working to overcome this by studying the feed process. Using the method of pumping suspension [99], nanocomposite-enhanced PLA biodegradable composites were obtained, which has better dispersibility. Another technique is wet extrusion [101]. Compared to melt extrusion, the wet extrusion has a lower temperature and is suitable for applying in biomedical applications. Because the temperature of the melt extrusion is too high to degrade the protein. At present, Danya M. Lavin’s team [102] used the wet extrusion method to prepare a self-assembled microfiber scaffold for drug delivery. The polylactic acid solution was added dropwise into a uniformly stirred, water-insoluble solvent to make it a liquid non-woven polymer. Fibrous scaffolds, then adjust the concentration of polymer spinning dope and increase the ratio of silicone oil to petroleum ether to achieve fiber diameter control.

#### 4.1.2. Processing of Nanoclay Based Bio-Nanocomposites

At present, nanoclay biodegradable composites are mainly mixed by means of intercalation layered silicate. There are three main methods: (1) polymer solution embedding; (2) in-situ polymerization; and (3) melt embedding method.

The so-called polymer solution embedding method mainly works by macromolecule clay intercalation solvent in the polymer [103]. By this way, the nanoclay can be embedded in the polymer and will not damage the internal structure of the polymer, forming nanoclay–polymer composites. However, this method requires a large amount of solvent, thereby displacing a large amount of waste liquid, which has an adverse effect on the environment.

In-situ polymerization utilizes the polymerization of monomers in phyllosilicates. In this method, the nanoclays expand in liquid monomer. Under the action of polymerization, the nanoclays are effectively embedded in the polymer, a process requiring catalyst initiation of polymerization. The catalyst can effectively make the nanoclay fixed in the inner layer of silicate without falling off [104].

**Figure 4:**
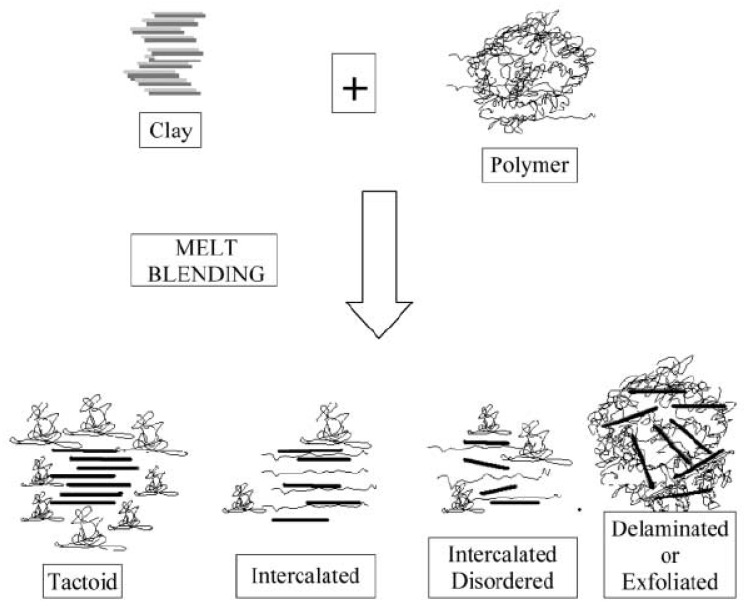
Schematic illustration of terminology used to describe nanocomposites formed from organoclays. Reproduced with permission from [106].

Melt embedding is currently the most widely used method in industrial production. When the temperature reaches the melting temperature, the nanoclay is annealed [105], in which case the polymer chains can enter the silicate interlayer, forming a sandwich structure. This method does not produce waste liquid as in the in-situ polymerization method and is a more economical and green method. This method can prepare various nanocomposites of different morphologies, depending on the manner in which the polymer chains are embedded and the types of functional groups. Figure 4 shows the formation of nanoclay composites.

#### 4.1.3. Processing of Polymer–Carbon Nanotubes Based Bio-Nanocomposites

Carbon nanotubes act as nanofillers to enhance biodegradable composites; the extent of their enhancement depends mainly on the molecular orientation and degree of dispersion. At present, single-walled carbon nanotubes (SWCNTs) and multi-walled carbon nanotubes (MWCNTs) are mainly dispersed in polymer composites, and van der Waals forces are used to enable the polymer matrix to condense carbon nanotubes. The degree of dispersion depends on the network structure of the structure. This uniformity depends on the molecular orientation of the matrix and its compatibility [107].

In addition, the differences in the size of the SWNTs and the larger surface energy lead to the polymerization of the MWCNTs. Therefore, it is difficult to uniformly distribute the surface of polymer. The current methods of uniformly dispersing carbon nanotubes include chemical modification [108], coating of carbon nanotubes [109], in-situ polymerization [104], ultrasonic dispersion [110], melt processing [111], addition of surfactants [112], electrospinning [113], electrochemistry and crystallization [114]. Uniform dispersion of carbon nanotubes can significantly increase the strength and toughness of composites, and improve the electrical conductivity of composites. It is an important measure for degradable biocomposites.

#### 4.1.4. Processing of Functional Nanocomposites

Hap-based nano-natural degradation of composite materials are used in medical applications. Hap composite materials can be prepared by traditional physicochemical methods [115]. The preparation method mainly adopts solvent casting. The solution concentration determines the degree of polymer dispersion while the mixing time and the mixing method determine polymer uniformity. Hap particles are not easily reacted due to the lack of reactive functional groups (hydroxyl groups). Currently, this problem can be solved by changing the Hap particle size and increasing the surface energy [116]. It should be noted that the catalyst added in the mix must be harmless and biocompatible without changing its properties.

**Figure 5:**
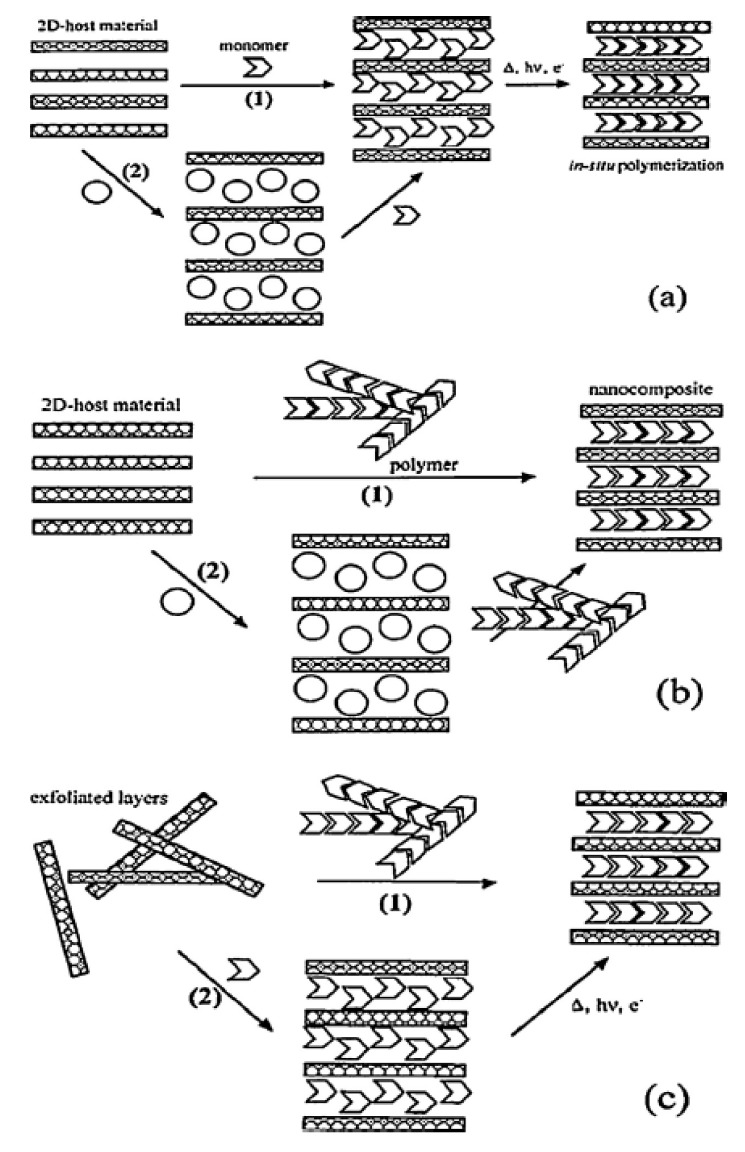
Preparation of LDH nanocomposites of various methods, (**a**) monomer exchange and in situ polymerization, (**b**) direct exchange, (**c**) exfoliated layers restacking. Reproduced with permission from [117].

The other is LDH nanocomposite. The polymer is mainly prepared by three methods: (a) monomer exchange and in situ polymerization; (b) coprecipitation or polymer displacement; and (c) polymer recombination [117]. Figure 5 shows three specific preparation methods. In-situ polymerization is to fill the interlayer of nanolayers with the reaction monomers, allowing them to polymerize between layers. The co-precipitation method refers to the fact that two or more cations are contained in a solution. They are present in a homogeneous solution and a precipitant is added. After the precipitation reaction, uniform precipitation of various components can be obtained. Polymer recombination refers to the process of reacting and orderly arranging different polymer monomers and reacting to form new polymers.

Method (a) is used in the preparation of polymer–LDH nanocomposites. The coprecipitation method is useful for layered hydroxide. The process consists of “co-assembly” synthesis of LDH in the presence of a polymer formed between LDH sheets [118].

### 4.2. Application

#### 4.2.1. Electronics

Nanofillers Enhanced Biodegradation Composites have been widely used in many fields and have many advanced developments in electronics, including diodes, solar cells, and electromagnetic applications [119]. With the continuous increase of electronic devices, abandoned electronic devices cause serious environmental pollution, and the appearance of biodegradable bio-composites greatly relieves the environmental pressure.

Nanocellulose bio-composites are widely used in medical, electronics, packaging and other fields. They are currently used in the development of flexible electronic equipment using roll-to-roll manufacturing process [120,121,122]. The technology relies on the substrate material and nanocellulose composite material as the base material to achieve the purpose of preparation. Masaya Nogi and co-workers [120,122] have experimentally proved the advantage of nanoscale reinforcing using cellulose nanofibers. They obtained transparent composites by enhancing various types of resin using BC nanofibers, even in the fiber content of up to 70 wt %. As BC nanofibers are bundles of semi-crystalline and extended cellulose chains, the obtained nanocomposites are not only highly transparent and flexible, but also present high mechanical strength comparable to low carbon steel and low coefficient of thermal expansion comparable to silicon, which make the composite suitable for applications. Moreover, they have succeeded in depositing an electroluminescent layer (comprised of organic light-emitting diodes) on these transparent BC nanocomposites. Petersson et al. [123] successfully prepared poly(lactic acid) cellulose-based biodegradable nanocomposites. They found that the poly(lactic acid) cellulose base can enhance the mechanical properties and thermal stability of the materials. It was concluded through experiments that the poly(lactic acid) cellulose-based biodegradable nanomaterials. The composite material is stable at 220 °C, so the material can be suitable for high temperature environments.

**Figure 6:**
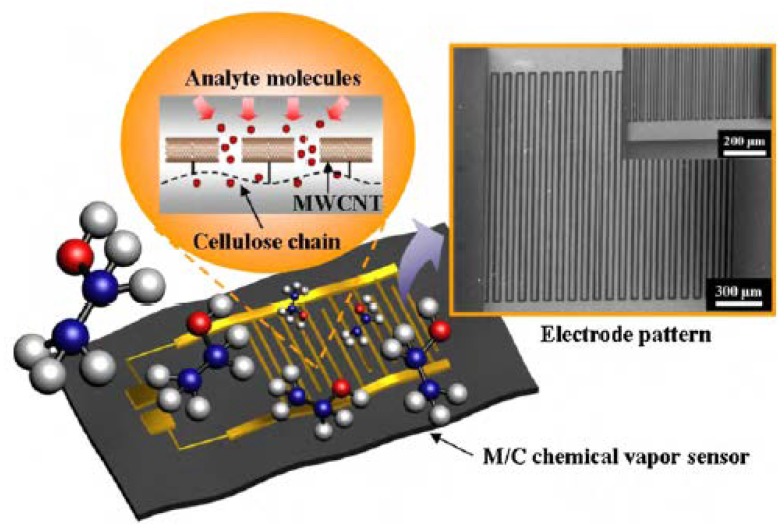
Schematic of chemical vapor sensor made with M/C paper and IDT shaped electrode (reproduced with permission [105]).

Carbon nanotube biodegradable composites have been applied in the development of flexible sensors. Sensors of this kind of materials can be applied in various temperature, humidity and complex chemical environments with better electromagnetic and mechanical properties [124,125]. Han and co-workers [124] reported a humidity sensor on cellulose paper using functionalized single-walled carbon nanotubes. The conductance displacement of the nanotube network wrapped on microfibril cellulose was used for humidity sensing. Compared with control sensors made on glass substrates, cellulose mediated charge transfer on paper enhances sensitivity. They furtherly prepared a similar CNT-based sensor device on cellulose paper for ammonia sensing [125]. At present, Yun et al. [105] manufactured carbon nanotube–cellulose biodegradable composites, which served as a base material for chemical vapor sensors. Cellulose solution was prepared by dissolving cotton pulp in LiCl/N, N-dimethylacetamide solution. The multi-walled carbon nanotubes (MWCNTs) were covalently grafted to cellulose by reacting imidazolides–MWCNTs with cellulose solution. Figure 6 below is a schematic diagram of a M/C paper and cross finger electrode chemical gas sensor. Its good economic benefits, biocompatibility and eco-friendly advantages led the sensor to receive extensive attention.

Carbon nanotube biodegradable composite materials are used in solar cells. Carbon nanotubes added to the flexible photovoltaic cells can directly improve the conductivity of the polymer and enhanced solar cell photon absorption capacity. Valentin et al. [126] developed a novel method of carbon nanotube and researched their electrical properties. It is concluded that the SWNTs composites can be used in organic conductive materials.

#### 4.2.2. Packaging Industries

There are three major drawbacks with biodegradable plastics currently used for packaging: performance, processing and cost. Emerging nanofillers enhance biodegradable composite materials to help overcome the above problems. Nanofilling can effectively improve these problems, and nanocomposites have significant advantages over traditional composites.

**Figure 7:**
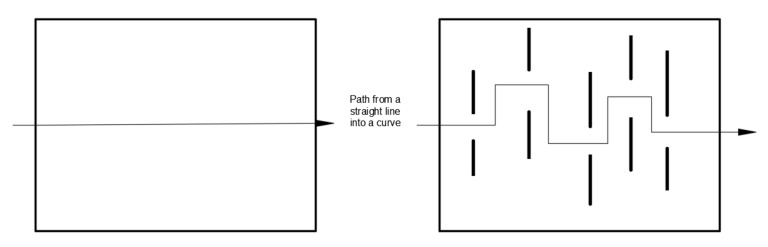
Nanoclay filler composite diffusion path schematic.

The materials currently used in food packaging mainly value their stretchability and permeability [127]. Nanofiller biodegradable composite materials not only have the key features of metal-based packaging, but also other good properties including mechanical properties, thermodynamic properties, environmental harm and so on. Furthermore, nanofiller composites may significantly improve the high barrier properties of polymers [104]. Due to the presence of nanofillers, the molecular pathways increase as it passes through the substrate. The presence of the nanoclay layer allows a significant increase in the diffusion path of gas or other water vapor molecules through the polymer, resulting in a substantial reduction in the rate of permeation through the polymer, thereby effectively enhancing its barrier properties. Figure 7 shows the nanoclay filler composite diffusion path schematic [128]. The figure shows that due to the presence of the nanoclay layer, the movement path of the water vapor molecules becomes longer, and the straight line from the beginning becomes a curve, which is why the nanoclay composite material improves the barrier properties.

Barrier properties of nanofiller biodegradable composites mainly depend on the molecular orientation in the polymer matrix material and the dispersion uniformity of the nanofiller. Comparing the various nanofillers, nanoclay has the best effect. Due to cost and ease of processing, PLA, PHB and starch-based nanocomposites are the most popular in the packaging industry [129].

As the most popular biodegradable material, PLA is involved in the preparation of many products such as cups, cutlery and packaging boxes [130]. PLA is mainly pressed into the cardboard by squeezing the coating layer, which is further developed and applied as a packaging material. Using PLA as a substrate material and nanoclay as a filler to enhance the performance of PLA, not only has the characteristics of PLA, but also enhances the barrier property of composites. Chang et al. [131] studied the effect of the nanoclay modification of PLA. The researchers prepared a melt-intercalated nanocomposite. The results show that the permeability of all nanocomposites decreases. In addition, the researchers also studied the effects of shear and feed rates on the permeability of PLA nanocomposite films. The oxygen barrier properties of PLA nanocomposites have been improved by 15–48% compared to that of pure PLA materials [132]. Moreover, the shear rate and feed rate have little effect on the forming, so the barrier properties of PLA nanocomposites depend mainly on the molecular orientation of the substrate material and the dispersion uniformity of the nanofiller.

For PHA to be able to enter the researcher’s field of vision depends mainly on its high hydrophobicity, as it is widely used in coatings and films [133]. In the food packaging, PHA barrier properties and polyethylene are very similar, which makes PHA useful in packaging materials. However, the workability and gas barrier properties of PHA have limited their development in the packaging field. Currently, Sanchez-Garcia et al. [134] studied the relationship between morphology and mechanical properties, including tensile strength and Young’s modulus. The high dispersion improves the barrier properties of PHB/clay nanocomposites.

Degradable bags made from starch-based substrates [135,136,137] result in many defects due to the high hydrophilicity of the starch, which can now be overcome using nanocomposites. Park et al. [138]. studied the permeability of different nanomaterials in water demonstrating that nanocomposites have an impediment to the penetration of water, which is in favor of the development of packaging technology.

Nanocomposites would lead to a variation in the permeability of water vapor, which is related to the saturation of the polymer base material and the nanofiller [139,140]. In most cases, different nanocomposites will have different degrees of saturation, thus resulting in different permeabilities. Moreover, the semi-crystalline polymer itself has crystalline regions and non-crystalline regions that are impermeable to molecules and therefore result in different permeabilities. In addition, the decrease in permeability is mainly due to the increase in the molecular path of the molecules through the polymer by the nanofillers, which is an effective way to increase the movement of gas molecules [141]. The effect of nanofillers on the permeability of the composite depends not only on the crystallinity of the substrate itself, but also on the different types of nanofillers.

#### 4.2.3. Medical Applications

Biodegradable composites rely on their biocompatibility and versatility and these materials have been widely used in the medical field. Biocompatible composite materials play a role in the human body without adversely affecting drugs. Therefore, bio-based composite materials have drawn more and more attention and biocompatible materials have been more clinically used [142,143,144,145,146,147]. In addition, soy-derived polymers have proven useful as bone fillers [148]. Bacterial nanocellulose has also been shown to be useful for artificial blood vessels [149]. Nanofiller biodegradable composite materials can be applied to clinical medicine.

Biodegradable nanocomposites are very useful in tissue engineering for the regeneration of primary tissue structures. Nanocomposites with three major characteristics can be applied to tissue engineering: (1) the extracellular matrix consisting of macromolecules; (2) the extracellular matrix where fiber forms exist; and (3) macromolecules in the extracellular matrix have a diameter of less than 500 nm [150].

The researchers found that the toxins in BNC can be easily eliminated by the use of sodium hydroxide treatment and water purification 13 purification methods [151]. Highly inhibited water in the fiber network prevents the adsorption of proteins in the blood, which is beneficial to blood compatibility. Bacterial nanofiber biodegradable composites with good fiber networks and pores provide a good environment for cell growth. This is a very important aspect of artificial blood vessels, proving that artificial blood vessels can form new blood vessel tissue in animal experiments.

Tang’s group [149] used two-photon reactor to prepare BNC composite artificial blood vessel. Researchers successfully prepared double-silicon (D-BNC) and single-silicon (S-BNC) artificial blood vessels, relying mainly on the unique properties of bacterial nanofillers. The researchers explained the process and nature of the biosynthesis of the artificial blood vessels. The results showed that the BNC artificial blood vessels rely on an ultra-fine, uniform fiber network and high porosity to be able to act as biomedical prostheses and to function in living organisms.

In addition, electrospun biocompatible polymer nanofiber composites are currently capable of performing hard tissue repairs in the form of porous membranes for implantation in humans [152]. Nanofiber PLA-PEG mosaic copolymer composites prepared by electrospinning can be used for bone tissue engineering scaffolds. Adding nanofiber fillers can not only increase the tensile strength and flexibility of the bones, but also increase the stiffness and strength of the bones. At present, the skeleton uses porous scaffold structure. This structure has high elasticity and plays a role in protecting the skeleton and has been successfully implanted in animal models to achieve bone regeneration [153].

## 5. Summary and Outlook

This article mainly introduces the research status of nanofillers to enhance biodegradable composite materials. This article describes different kinds of nanofillers and biodegradable polymers for biodegradable nanocomposites. Different nanofillers have different treatment methods and apply to different fields. Biodegradable composite materials use nanofillers to enhance performance and these biodegradable nanocomposites have broader application prospects. This paper introduces the enhancement of bio-based polymers by nanoclay, nanocellulose, carbon nanotubes and functional nanofillers, which improves the deficiencies of bio-based polymers. The process of adding fillers involves a series of different treatment methods, mainly by solvent casting and melt processing methods, which can be applied to nanocellulose. The method of polymer intercalation is mainly suitable for the addition of nanoclay filler. For carbon nanotubes, the current industry mainly uses the melt extrusion method.

Biodegradable nanocomposites have mainly been prepared for use in electronics, packaging and biomedical applications. Currently, in electronics, nanocellulose composite materials can be applied to the preparation of electronic displays, flexible sensors, light-emitting diodes, etc., to promote the development of flexible electronic devices. In the field of packaging, biodegradable polymers exist to fix three major defects: performance, processing and cost. The nanofiller enhanced biodegradable composite materials can overcome the above problems, and help to improve the performance of biodegradable polymers. In biomedicine, nano-enhanced biodegradable composite materials are mainly used in the field of tissue engineering, drug delivery and gene therapy. Bio-composites rely on their biocompatibility and has been the concern of researchers. The use of nanofillers has been explored to enhance bio-based composite materials to obtain more functions and achieve more medical breakthroughs.

Nanofiller reinforced biodegradable composite materials are more widely used, and the area has attracted many researchers. In future research, researchers are more likely to focus on nanofillers to enhance composite processes, and to study more highly industrialized and efficient processes that are very difficult for this nanotechnology. In the future, biodegradable composite materials can replace most of the current materials, which is very important for sustaining our life; therefore, it is an urgent task to study nanofillers to enhance biodegradable composite materials.

## References

[1] Sridhar V., Lee I., Chun H.H., Park H. (2013). Graphene reinforced biodegradable poly(3-hydroxybutyrate-co-4-hydroxybutyrate) nano-composites. Express Polym. Lett..

[2] Fan H., Wang L., Zhao K., Li N., Shi Z., Ge Z., Jin Z. (2010). Fabrication, Mechanical Properties, and Biocompatibility of Graphene-Reinforced Chitosan Composites. Biomacromolecules.

[3] Mathew A.P., Oksman K., Sain M. (2005). Mechanical properties of biodegradable composites from poly lactic acid (PLA) and microcrystalline cellulose (MCC). J. Appl. Polym. Sci..

[4] Wang X., Yang H., Song L., Hu Y., Xing W., Lu H. (2011). Morphology, mechanical and thermal properties of graphene-reinforced poly(butylene succinate) nanocomposites. Compos. Sci. Technol..

[5] Liu H., Li J., Ren N., Qiu J., Mou X. (2013). Graphene oxide-reinforced biodegradable genipin-cross-linked chitosan fluorescent biocomposite film and its cytocompatibility. Int. J. Nanomed..

[6] Lalwani G., Henslee A.M., Farshid B., Lin L., Kasper F.K., Qin Y.-X., Mikos A.G., Sitharaman B. (2013). Two-Dimensional Nanostructure-Reinforced Biodegradable Polymeric Nanocomposites for Bone Tissue Engineering. Biomacromolecules.

[7] Jia W., Gong R.H., Hogg P.J. (2014). Poly (lactic acid) fibre reinforced biodegradable composites. Compos. Part B Eng..

[8] Nazhat S.N., Kellomäki M., Törmälä P., Tanner K.E., Bonfield W. (2001). Dynamic mechanical characterization of biodegradable composites of hydroxyapatite and polylactides. J. Biomed. Mater. Res..

[9] Nam T.H., Ogihara S., Tung N.H., Kobayashi S. (2011). Effect of alkali treatment on interfacial and mechanical properties of coir fiber reinforced poly(butylene succinate) biodegradable composites. Compos. Part B Eng..

[10] Barkoula N.M., Garkhail S.K., Peijs T. (2010). Biodegradable composites based on flax/polyhydroxybutyrate and its copolymer with hydroxyvalerate. Ind. Crops Prod..

[11] Akil H.M., Omar M.F., Mazuki A.A.M., Safiee S., Ishak Z.A.M., Abu Bakar A. (2011). Kenaf fiber reinforced composites: A review. Mater. Des..

[12] La Mantia F.P., Morreale M. (2011). Green composites: A brief review. Compos. Part A Appl. Sci. Manuf..

[13] Abdul Khalil H.P.S., Bhat A.H., Ireana Yusra A.F. (2012). Green composites from sustainable cellulose nanofibrils: A review. Carbohydr. Polym..

[14] Koronis G., Silva A., Fontul M. (2013). Green composites: A review of adequate materials for automotive applications. Compos. Part B Eng..

[15] Saba N., Tahir P., Jawaid M. (2014). A Review on Potentiality of Nano Filler/Natural Fiber Filled Polymer Hybrid Composites. Polymers.

[16] Chieng B., Ibrahim N., Yunus W., Hussein M., Then Y., Loo Y. (2014). Effects of Graphene Nanoplatelets and Reduced Graphene Oxide on Poly(lactic acid) and Plasticized Poly(lactic acid): A Comparative Study. Polymers.

[17] Yusoff R.B., Takagi H., Nakagaito A.N. (2016). Tensile and flexural properties of polylactic acid-based hybrid green composites reinforced by kenaf, bamboo and coir fibers. Ind. Crops Prod..

[18] Hamad K., Kaseem M., Ko Y.G., Deri F. (2014). Biodegradable polymer blends and composites: An overview. Polym. Sci. Ser. A.

[19] Chen G.-Q., Patel M.K. (2011). Plastics Derived from Biological Sources: Present and Future: A Technical and Environmental Review. Chem. Rev..

[20] Carvalho R.A., Santos T.A., de Azevedo V.M., Felix P.H.C., Dias M.V., Borges S.V. (2018). Bio-nanocomposites for food packaging applications: Effect of cellulose nanofibers on morphological, mechanical, optical and barrier properties. Polym. Int..

[21] Siqueira G., Bras J., Dufresne A. (2010). Cellulosic Bionanocomposites: A Review of Preparation, Properties and Applications. Polymers.

[22] Pracella M., Haque M.M.-U., Puglia D. (2014). Morphology and properties tuning of PLA/cellulose nanocrystals bio-nanocomposites by means of reactive functionalization and blending with PVAc. Polymer.

[23] Chen Z., Zhang A., Wang X., Zhu J., Fan Y., Yu H., Yang Z. (2017). The Advances of Carbon Nanotubes in Cancer Diagnostics and Therapeutics. J. Nanomater..

[24] Chen Z., Zhang A., Yang Z., Wang X., Chang L., Chen Z., James Lee L. (2016). Application of DODMA and Derivatives in Cationic Nanocarriers for Gene Delivery. Curr. Org. Chem..

[25] Xie J., Yang Z., Zhou C., Zhu J., Lee R.J., Teng L. (2016). Nanotechnology for the delivery of phytochemicals in cancer therapy. Biotechnol. Adv..

[26] Mahalik N.P., Nambiar A.N. (2010). Trends in food packaging and manufacturing systems and technology. Trends Food Sci. Technol..

[27] Mihindukulasuriya S.D.F., Lim L.T. (2014). Nanotechnology development in food packaging: A review. Trends Food Sci. Technol..

[28] Bugnicourt E., Cinelli P., Lazzeri A., Alvarez V. (2014). Polyhydroxyalkanoate (PHA): Review of synthesis, characteristics, processing and potential applications in packaging. Express Polym. Lett..

[29] Ahmed J., Varshney S.K. (2011). Polylactides—Chemistry, Properties and Green Packaging Technology: A Review. Int. J. Food Prop..

[30] Barlow C.Y., Morgan D.C. (2013). Polymer film packaging for food: An environmental assessment. Resour. Conserv. Recycl..

[31] Rhim J.-W., Park H.-M., Ha C.-S. (2013). Bio-nanocomposites for food packaging applications. Prog. Polym. Sci..

[32] Othman S.H. (2014). Bio-nanocomposite Materials for Food Packaging Applications: Types of Biopolymer and Nano-sized Filler. Agric. Agric. Sci. Procedia.

[33] Busolo M.A., Fernandez P., Ocio M.J., Lagaron J.M. (2010). Novel silver-based nanoclay as an antimicrobial in polylactic acid food packaging coatings. Food Addit. Contam. Part A.

[34] Huang J.-Y., Li X., Zhou W. (2015). Safety assessment of nanocomposite for food packaging application. Trends Food Sci. Technol..

[35] Cherpinski A., Torres-Giner S., Cabedo L., Méndez J.A., Lagaron J.M. (2018). Multilayer structures based on annealed electrospun biopolymer coatings of interest in water and aroma barrier fiber-based food packaging applications. J. Appl. Polym. Sci..

[36] Peelman N., Ragaert P., De Meulenaer B., Adons D., Peeters R., Cardon L., Van Impe F., Devlieghere F. (2013). Application of bioplastics for food packaging. Trends Food Sci. Technol..

[37] Chen Z., Cong M., Hu J., Yang Z., Chen Z. (2016). Preparation of Functionalized TiO_2_ Nanotube Arrays and Their Applications. Sci. Adv. Mater..

[38] Kuang T., Fu D., Chang L., Yang Z., Yang J., Fan P., Zhong M., Chen F., Peng X. (2016). Enhanced Photocatalysis of Yittium-Doped TiO_2_/D-PVA Composites: Degradation of Methyl Orange (MO) and PVC Film. Sci. Adv. Mater..

[39] Dong Y., Zhou G., Chen J., Shen L., Jianxin Z., Xu Q., Zhu Y. (2016). A new LED device used for photodynamic therapy in treatment of moderate to severe acne vulgaris. Photodiagn. Photodyn. Ther..

[40] Sha L., Chen Z., Chen Z., Zhang A., Yang Z. (2016). Polylactic Acid Based Nanocomposites: Promising Safe and Biodegradable Materials in Biomedical Field. Int. J. Polym. Sci..

[41] Chen Z., Chen Z., Yang Z., Hu J., Yang Y., Chang L., Lee L.J., Xu T. (2015). Preparation and characterization of vacuum insulation panels with super-stratified glass fiber core material. Energy.

[42] Thakur V.K., Thakur M.K., Raghavan P., Kessler M.R. (2014). Progress in Green Polymer Composites from Lignin for Multifunctional Applications: A Review. ACS Sustain. Chem. Eng..

[43] Frone A.N., Berlioz S., Chailan J.-F., Panaitescu D.M. (2013). Morphology and thermal properties of PLA–cellulose nanofibers composites. Carbohydr. Polym..

[44] Georgiopoulos P., Kontou E., Niaounakis M. (2013). Thermomechanical properties and rheological behavior of biodegradable composites. Polym. Compos..

[45] Wu G., Deng X., Song J., Chen F. (2018). Enhanced biological properties of biomimetic apatite fabricated polycaprolactone/chitosan nanofibrous bio-composite for tendon and ligament regeneration. J. Photochem. Photobiol. B Biol..

[46] Wang P., Zhang D., Zhou Y., Li Y., Fang H., Wei H., Ding Y. (2017). A well-defined biodegradable 1,2,3-triazolium-functionalized PEG-b-PCL block copolymer: Facile synthesis and its compatibilization for PLA/PCL blends. Ionics.

[47] Iwata T. (2015). Biodegradable and Bio-Based Polymers: Future Prospects of Eco-Friendly Plastics. Angew. Chem. Int. Ed..

[48] Satoh K. (2015). Controlled/living polymerization of renewable vinyl monomers into bio-based polymers. Polym. J..

[49] Okuda T., Ishimoto K., Ohara H., Kobayashi S. (2012). Renewable Biobased Polymeric Materials: Facile Synthesis of Itaconic Anhydride-Based Copolymers with Poly(l-lactic acid) Grafts. Macromolecules.

[50] Nakajima H., Dijkstra P., Loos K. (2017). The Recent Developments in Biobased Polymers toward General and Engineering Applications: Polymers that are Upgraded from Biodegradable Polymers, Analogous to Petroleum-Derived Polymers, and Newly Developed. Polymers.

[51] Lotz B., Li G., Chen X., Puiggali J. (2017). Crystal polymorphism of polylactides and poly(Pro-alt-CO): The metastable beta and gamma phases. Formation of homochiral PLLA phases in the PLLA/PDLA blends. Polymer.

[52] Kanetaka Y., Yamazaki S., Kimura K. (2016). Preparation of poly(ether ketone)s derived from 2,5-furandicarboxylic acid via nucleophilic aromatic substitution polymerization. J. Polym. Sci. Part A Polym. Chem..

[53] Mochizuki M. (2010). Crystallization Behaviors of highly LLA-rich PLA Effects of D-isomer ratio of PLA on the rate of crystallization, crystallinity, and melting point. Sen-I Gakkaishi.

[54] Reddy M.M., Vivekanandhan S., Misra M., Bhatia S.K., Mohanty A.K. (2013). Biobased plastics and bionanocomposites: Current status and future opportunities. Prog. Polym. Sci..

[55] Guo Y., Chang C.-C., Halada G., Cuiffo M.A., Xue Y., Zuo X., Pack S., Zhang L., He S., Weil E. (2017). Engineering flame retardant biodegradable polymer nanocomposites and their application in 3D printing. Polym. Degrad. Stab..

[56] Du Y., Wu T., Yan N., Kortschot M.T., Farnood R. (2014). Fabrication and characterization of fully biodegradable natural fiber-reinforced poly(lactic acid) composites. Compos. Part B Eng..

[57] Lim L.T., Auras R., Rubino M. (2008). Processing technologies for poly(lactic acid). Prog. Polym. Sci..

[58] Thummarungsan N., Paradee N., Pattavarakorn D., Sirivat A. (2018). Influence of graphene on electromechanical responses of plasticized poly(lactic acid). Polymer.

[59] Hamad K., Kaseem M., Yang H.W., Deri F., Ko Y.G. (2015). Properties and medical applications of polylactic acid: A review. Express Polym. Lett..

[60] Insomphun C., Chuah J.-A., Kobayashi S., Fujiki T., Numata K. (2016). Influence of Hydroxyl Groups on the Cell Viability of Polyhydroxyalkanoate (PHA) Scaffolds for Tissue Engineering. ACS Biomater. Sci. Eng..

[61] Madison L.L., Huisman G.W. (1999). Metabolic engineering of poly(3-hydroxyalkanoates): From DNA to plastic. Microbiol. Mol. Biol. Rev. MMBR.

[62] Chen G.-Q., Jiang X.-R., Guo Y. (2016). Synthetic biology of microbes synthesizing polyhydroxyalkanoates (PHA). Synth. Syst. Biotechnol..

[63] Lim J., You M., Li J., Li Z. (2017). Emerging bone tissue engineering via Polyhydroxyalkanoate (PHA)-based scaffolds. Mater. Sci. Eng. C.

[64] Zhang J., Shishatskaya E.I., Volova T.G., da Silva L.F., Chen G.-Q. (2018). Polyhydroxyalkanoates (PHA) for therapeutic applications. Mater. Sci. Eng. C.

[65] Chen G.-Q., Jiang X.-R. (2017). Engineering bacteria for enhanced polyhydroxyalkanoates (PHA) biosynthesis. Synth. Syst. Biotechnol..

[66] Korkakaki E., van Loosdrecht M.C.M., Kleerebezem R. (2017). Impact of phosphate limitation on PHA production in a feast-famine process. Water Res..

[67] Koller M., Maršálek L., de Sousa Dias M.M., Braunegg G. (2017). Producing microbial polyhydroxyalkanoate (PHA) biopolyesters in a sustainable manner. New Biotechnol..

[68] Burniol-Figols A., Varrone C., Daugaard A.E., Le S.B., Skiadas I.V., Gavala H.N. (2018). Polyhydroxyalkanoates (PHA) production from fermented crude glycerol: Study on the conversion of 1,3-propanediol to PHA in mixed microbial consortia. Water Res..

[69] Kovalcik A., Meixner K., Mihalic M., Zeilinger W., Fritz I., Fuchs W., Kucharczyk P., Stelzer F., Drosg B. (2017). Characterization of polyhydroxyalkanoates produced by Synechocystis salina from digestate supernatant. Int. J. Biol. Macromol..

[70] Chin K.-M., Sung Ting S., Ong H.L., Omar M. (2018). Surface functionalized nanocellulose as a veritable inclusionary material in contemporary bioinspired applications: A review. J. Appl. Polym. Sci..

[71] Scherner M., Reutter S., Klemm D., Sterner-Kock A., Guschlbauer M., Richter T., Langebartels G., Madershahian N., Wahlers T., Wippermann J. (2014). In vivo application of tissue-engineered blood vessels of bacterial cellulose as small arterial substitutes: Proof of concept?. J. Surg. Res..

[72] Lin N., Dufresne A. (2014). Nanocellulose in biomedicine: Current status and future prospect. Eur. Polym. J..

[73] Abitbol T., Rivkin A., Cao Y., Nevo Y., Abraham E., Ben-Shalom T., Lapidot S., Shoseyov O. (2016). Nanocellulose, a tiny fiber with huge applications. Curr. Opin. Biotechnol..

[74] Akhavan Mahdavi S., Mahdi Jafari S., Assadpoor E., Dehnad D. (2016). Microencapsulation optimization of natural anthocyanins with maltodextrin, gum Arabic and gelatin. Int. J. Biol. Macromol..

[75] Kuo P.-Y., Barros L.d.A., Yan N., Sain M., Qing Y., Wu Y. (2017). Nanocellulose composites with enhanced interfacial compatibility and mechanical properties using a hybrid-toughened epoxy matrix. Carbohydr. Polym..

[76] Tardy B.L., Yokota S., Ago M., Xiang W., Kondo T., Bordes R., Rojas O.J. (2017). Nanocellulose–surfactant interactions. Curr. Opin. Colloid Interface Sci..

[77] Lee K.-Y., Aitomäki Y., Berglund L.A., Oksman K., Bismarck A. (2014). On the use of nanocellulose as reinforcement in polymer matrix composites. Compos. Sci. Technol..

[78] Mishra R.K., Sabu A., Tiwari S.K. (2018). Materials chemistry and the futurist eco-friendly applications of nanocellulose: Status and prospect. J. Saudi Chem. Soc..

[79] Chivrac F., Pollet E., Avérous L. (2009). Progress in nano-biocomposites based on polysaccharides and nanoclays. Mater. Sci. Eng. R Rep..

[80] Majeed K., Jawaid M., Hassan A., Abu Bakar A., Abdul Khalil H.P.S., Salema A.A., Inuwa I. (2013). Potential materials for food packaging from nanoclay/natural fibres filled hybrid composites. Mater. Des..

[81] Malin F., Znoj B., Šegedin U., Skale S., Golob J., Venturini P. (2013). Polyacryl–nanoclay composite for anticorrosion application. Prog. Org. Coat..

[82] Hakamy A., Shaikh F.U.A., Low I.M. (2014). Characteristics of hemp fabric reinforced nanoclay–cement nanocomposites. Cem. Concr. Compos..

[83] Felbeck T., Bonk A., Kaup G., Mundinger S., Grethe T., Rabe M., Vogt U., Kynast U. (2016). Porous nanoclay polysulfone composites: A backbone with high pore accessibility for functional modifications. Microporous Mesoporous Mater..

[84] Shettar M., Achutha Kini U., Sharma S.S., Hiremath P. (2017). Study on Mechanical Characteristics of Nanoclay Reinforced Polymer Composites. Mater. Today Proc..

[85] Memiş S., Tornuk F., Bozkurt F., Durak M.Z. (2017). Production and characterization of a new biodegradable fenugreek seed gum based active nanocomposite film reinforced with nanoclays. Int. J. Biol. Macromol..

[86] Daenen M., Zhang L., Erni R., Williams O.A., Hardy A., Van Bael M.K., Wagner P., Haenen K., Nesladek M., Van Tendeloo G. (2009). Diamond Nucleation by Carbon Transport from Buried Nanodiamond TiO_2_ Sol-Gel Composites. Adv. Mater..

[87] Ma P., Jiang L., Ye T., Dong W., Chen M. (2014). Melt Free-Radical Grafting of Maleic Anhydride onto Biodegradable Poly(lactic acid) by Using Styrene as A Comonomer. Polymers.

[88] Hapuarachchi T.D., Peijs T. (2010). Multiwalled carbon nanotubes and sepiolite nanoclays as flame retardants for polylactide and its natural fibre reinforced composites. Compos. Part A Appl. Sci. Manuf..

[89] Tan B., Thomas N.L. (2016). A review of the water barrier properties of polymer/clay and polymer/graphene nanocomposites. J. Membr. Sci..

[90] Young R.J., Kinloch I.A., Gong L., Novoselov K.S. (2012). The mechanics of graphene nanocomposites: A review. Compos. Sci. Technol..

[91] Sherlala A.I.A., Raman A.A.A., Bello M.M., Asghar A. (2018). A review of the applications of organo-functionalized magnetic graphene oxide nanocomposites for heavy metal adsorption. Chemosphere.

[92] Kashi S., Gupta R.K., Baum T., Kao N., Bhattacharya S.N. (2016). Dielectric properties and electromagnetic interference shielding effectiveness of graphene-based biodegradable nanocomposites. Mater. Des..

[93] Baruah P., Karak N. (2016). Bio-based tough hyperbranched epoxy/graphene oxide nanocomposite with enhanced biodegradability attribute. Polym. Degrad. Stab..

[94] Hule R.A., Pochan D.J. (2011). Polymer Nanocomposites for Biomedical Applications. MRS Bulletin.

[95] Millon L.E., Wan W.K. (2006). The polyvinyl alcohol–bacterial cellulose system as a new nanocomposite for biomedical applications. J. Biomed. Mater. Res. Part B Appl. Biomater..

[96] Bhatia S.K., Kurian J.V. (2007). Biological characterization of Sorona polymer from corn-derived 1,3-propanediol. Biotechnol. Lett..

[97] Ramadas M., Bharath G., Ponpandian N., Ballamurugan A.M. (2017). Investigation on biophysical properties of Hydroxyapatite/Graphene oxide (HAp/GO) based binary nanocomposite for biomedical applications. Mater. Chem. Phys..

[98] Dufresne A. (2010). Processing of Polymer Nanocomposites Reinforced with Polysaccharide Nanocrystals. Molecules.

[99] Oksman K., Mathew A.P., Bondeson D., Kvien I. (2006). Manufacturing process of cellulose whiskers/polylactic acid nanocomposites. Compos. Sci. Technol..

[100] Bondeson D., Oksman K. (2007). Polylactic acid/cellulose whisker nanocomposites modified by polyvinyl alcohol. Compos. Part A Appl. Sci. Manuf..

[101] Xia Y., Shi C.-Y., Fang J.-G., Wang W.-Q. (2016). Approaches to developing fast release pellets via wet extrusion-spheronization. Pharm. Dev. Technol..

[102] Lavin D.M., Harrison M.W., Tee L.Y., Wei K.A., Mathiowitz E. (2012). A novel wet extrusion technique to fabricate self-assembled microfiber scaffolds for controlled drug delivery. J. Biomed. Mater. Res. Part A.

[103] Vaia R.A., Giannelis E.P. (1997). Polymer Melt Intercalation in Organically-Modified Layered Silicates: Model Predictions and Experiment. Macromolecules.

[104] Sinha Ray S., Okamoto M. (2003). Polymer/layered silicate nanocomposites: A review from preparation to processing. Prog. Polym. Sci..

[105] Yun S., Kim J. (2010). Multi-walled carbon nanotubes–cellulose paper for a chemical vapor sensor. Sens. Actuators B Chem..

[106] Dennis H.R., Hunter D.L., Chang D., Kim S., White J.L., Cho J.W., Paul D.R. (2001). Effect of melt processing conditions on the extent of exfoliation in organoclay-based nanocomposites. Polymer.

[107] Shaffer M.S.P., Windle A.H. (1999). Analogies between Polymer Solutions and Carbon Nanotube Dispersions. Macromolecules.

[108] Eitan A., Jiang K., Dukes D., Andrews R., Schadler L.S. (2003). Surface Modification of Multiwalled Carbon Nanotubes: Toward the Tailoring of the Interface in Polymer Composites. Chem. Mater..

[109] Star A., Stoddart J.F., Steuerman D., Diehl M., Boukai A., Wong E.W., Yang X., Chung S.-W., Choi H., Heath J.R. (2001). Preparation and Properties of Polymer-Wrapped Single-Walled Carbon Nanotubes. Angew. Chem. Int. Ed..

[110] Qian D., Dickey E.C., Andrews R., Rantell T. (2000). Load transfer and deformation mechanisms in carbon nanotube-polystyrene composites. Appl. Phys. Lett..

[111] Siochi E.J., Working D.C., Park C., Lillehei P.T., Rouse J.H., Topping C.C., Bhattacharyya A.R., Kumar S. (2004). Melt processing of SWCNT-polyimide nanocomposite fibers. Compos. Part B Eng..

[112] Schadler L.S., Giannaris S.C., Ajayan P.M. (1998). Load transfer in carbon nanotube epoxy composites. Appl. Phys. Lett..

[113] Dror Y., Salalha W., Khalfin R.L., Cohen Y., Yarin A.L., Zussman E. (2003). Carbon Nanotubes Embedded in Oriented Polymer Nanofibers by Electrospinning. Langmuir.

[114] Chen G.Z., Shaffer M.S.P., Coleby D., Dixon G., Zhou W., Fray D.J., Windle A.H. (2000). Carbon Nanotube and Polypyrrole Composites Coating and Doping. Adv. Mater..

[115] Šupová M. (2009). Problem of hydroxyapatite dispersion in polymer matrices: A review. J. Mater. Sci. Mater. Med..

[116] Ahn E.S., Gleason N.J., Nakahira A., Ying J.Y. (2001). Nanostructure Processing of Hydroxyapatite-based Bioceramics. Nano Lett..

[117] Leroux F., Besse J.-P. (2001). Polymer Interleaved Layered Double Hydroxide: A New Emerging Class of Nanocomposites. Chem. Mater..

[118] Darder M., López-Blanco M., Aranda P., Leroux F., Ruiz-Hitzky E. (2005). Bio-Nanocomposites Based on Layered Double Hydroxides. Chem. Mater..

[119] Jung Y.J., Kar S., Talapatra S., Soldano C., Viswanathan G., Li X., Yao Z., Ou F.S., Avadhanula A., Vajtai R. (2006). Aligned Carbon Nanotube−Polymer Hybrid Architectures for Diverse Flexible Electronic Applications. Nano Lett..

[120] Nogi M., Yano H. (2008). Transparent Nanocomposites Based on Cellulose Produced by Bacteria Offer Potential Innovation in the Electronics Device Industry. Adv. Mater..

[121] Eichhorn S.J., Dufresne A., Aranguren M., Marcovich N.E., Capadona J.R., Rowan S.J., Weder C., Thielemans W., Roman M., Renneckar S. (2009). Review: Current international research into cellulose nanofibres and nanocomposites. J. Mater. Sci..

[122] Yano H., Sugiyama J., Nakagaito A.N., Nogi M., Matsuura T., Hikita M., Handa K. (2005). Optically Transparent Composites Reinforced with Networks of Bacterial Nanofibers. Adv. Mater..

[123] Petersson L., Kvien I., Oksman K. (2007). Structure and thermal properties of poly(lactic acid)/cellulose whiskers nanocomposite materials. Compos. Sci. Technol..

[124] Han J.-W., Kim B., Li J., Meyyappan M. (2012). Carbon Nanotube Based Humidity Sensor on Cellulose Paper. J. Phys. Chem. C.

[125] Han J.-W., Kim B., Li J., Meyyappan M. (2014). A carbon nanotube based ammonia sensor on cellulose paper. RSC Adv..

[126] Valentini L., Kenny J.M. (2005). Novel approaches to developing carbon nanotube based polymer composites: Fundamental studies and nanotech applications. Polymer.

[127] Johannson C. (2011). Bio-nanocomposites for food packaging applications. Nanocomposites with Biodegradable Polymers: Synthesis, Properties and Future Perspectives.

[128] Bharadwaj R.K. (2001). Modeling the Barrier Properties of Polymer-Layered Silicate Nanocomposites. Macromolecules.

[129] Azeredo H.M.C., Mattoso L.H.C., Wood D., Williams T.G., Avena-Bustillos R.J., McHugh T.H. (2009). Nanocomposite Edible Films from Mango Puree Reinforced with Cellulose Nanofibers. J. Food Sci..

[130] Auras R.A., Singh S.P., Singh J.J. (2005). Evaluation of oriented poly(lactide) polymers vs. existing PET and oriented PS for fresh food service containers. Packag. Technol. Sci..

[131] Chang J.-H., An Y.U., Sur G.S. (2003). Poly(lactic acid) nanocomposites with various organoclays. I. Thermomechanical properties, morphology, and gas permeability. J. Polym. Sci. Part B Polym. Phys..

[132] Thellen C., Orroth C., Froio D., Ziegler D., Lucciarini J., Farrell R., D’Souza N.A., Ratto J.A. (2005). Influence of montmorillonite layered silicate on plasticized poly(l-lactide) blown films. Polymer.

[133] Petersen K., Væggemose Nielsen P., Bertelsen G., Lawther M., Olsen M.B., Nilsson N.H., Mortensen G. (1999). Potential of biobased materials for food packaging. Trends Food Sci. Technol..

[134] Sanchez-Garcia M.D., Lagaron J.M. (2010). Novel clay-based nanobiocomposites of biopolyesters with synergistic barrier to UV light, gas, and vapour. J. Appl. Polym. Sci..

[135] Versino F., Lopez O.V., Garcia M.A., Zaritzky N.E. (2016). Starch-based films and food coatings: An overview. Starch Stärke.

[136] López O.V., Castillo L.A., García M.A., Villar M.A., Barbosa S.E. (2015). Food packaging bags based on thermoplastic corn starch reinforced with talc nanoparticles. Food Hydrocoll..

[137] Ibrahim H., Farag M., Megahed H., Mehanny S. (2014). Characteristics of starch-based biodegradable composites reinforced with date palm and flax fibers. Carbohydr. Polym..

[138] Park H.-M., Lee W.-K., Park C.-Y., Cho W.-J., Ha C.-S. (2003). Environmentally friendly polymer hybrids Part I Mechanical, thermal, and barrier properties of thermoplastic starch/clay nanocomposites. J. Mater. Sci..

[139] Gorrasi G., Pantani R., Murariu M., Dubois P. (2014). PLA/Halloysite Nanocomposite Films: Water Vapor Barrier Properties and Specific Key Characteristics. Macromol. Mater. Eng..

[140] Liu S., Cai P., Li X., Chen L., Li L., Li B. (2016). Effect of film multi-scale structure on the water vapor permeability in hydroxypropyl starch (HPS)/Na-MMT nanocomposites. Carbohydr. Polym..

[141] Chang P.R., Jian R., Yu J., Ma X. (2010). Fabrication and characterisation of chitosan nanoparticles/plasticised-starch composites. Food Chem..

[142] Yang Z., Xie J., Zhu J., Kang C., Chiang C., Wang X., Wang X., Kuang T., Chen F., Chen Z. (2016). Functional exosome-mimic for delivery of siRNA to cancer: In vitro and in vivo evaluation. J. Controll. Release.

[143] Yang Z., Chang L., Chiang C.-l., James Lee L. (2015). Micro-/nano-electroporation for active gene delivery. Curr. Pharm. Des..

[144] Yang Z., Chang L., Li W., Xie J. (2015). Novel biomaterials and biotechnology for nanomedicine. Eur. J. BioMed. Res..

[145] Zhou C., Yang Z., Teng L. (2014). Nanomedicine based on Nucleic Acids: Pharmacokinetic and Pharmacodynamic Perspectives. Curr. Pharm. Biotechnol..

[146] Yang Z., Yu B., Zhu J., Huang X., Xie J., Xu S., Yang X., Wang X., Yung B.C., Lee L.J. (2014). A microfluidic method to synthesize transferrin-lipid nanoparticles loaded with siRNA LOR-1284 for therapy of acute myeloid leukemia. Nanoscale.

[147] Xie J., Teng L., Yang Z., Zhou C., Liu Y., Yung B.C., Lee R.J. (2013). A Polyethylenimine-Linoleic Acid Conjugate for Antisense Oligonucleotide Delivery. BioMed. Res. Int..

[148] Giavaresi G., Fini M., Salvage J., Nicoli Aldini N., Giardino R., Ambrosio L., Nicolais L., Santin M. (2009). Bone regeneration potential of a soybean-based filler: Experimental study in a rabbit cancellous bone defects. J. Mater. Sci. Mater. Med..

[149] Tang J., Li X., Bao L., Chen L., Hong F.F. (2017). Comparison of two types of bioreactors for synthesis of bacterial nanocellulose tubes as potential medical prostheses including artificial blood vessels. J. Chem. Technol. Biotechnol..

[150] McCullen S.D., Ramaswamy S., Clarke L.I., Gorga R.E. (2009). Nanofibrous composites for tissue engineering applications. Wiley Interdiscip. Rev. Nanomed. Nanobiotechnol..

[151] Bodin A., Bäckdahl H., Fink H., Gustafsson L., Risberg B., Gatenholm P. (2007). Influence of cultivation conditions on mechanical and morphological properties of bacterial cellulose tubes. Biotechnol. Bioeng..

[152] Feng C., Khulbe K.C., Matsuura T. (2010). Recent progress in the preparation, characterization, and applications of nanofibers and nanofiber membranes via electrospinning/interfacial polymerization. J. Appl. Polym. Sci..

[153] Shi M., Yang R., Li Q., Lv K., Miron R.J., Sun J., Li M., Zhang Y. (2018). Inorganic Self-Assembled Bioactive Artificial Proto-Osteocells Inducing Bone Regeneration. ACS Appl. Mater. Interfaces.

